# Life Style Intervention Improves Retinopathy Status—The Finnish Diabetes Prevention Study

**DOI:** 10.3390/nu11071691

**Published:** 2019-07-23

**Authors:** A. Aro, A. Kauppinen, N. Kivinen, T. Selander, K. Kinnunen, J. Tuomilehto, S. Keinänen-Kiukaanniemi, J. Lindström, M. Uusitupa, K. Kaarniranta

**Affiliations:** 1 Department of Ophthalmology, Karelia Central Hospital, 80210 Joensuu, Finland; 2 School of Pharmacy, University of Eastern Finland, 70211 Kuopio, Finland; 3 Department of Ophthalmology, Kuopio University Hospital, 70029 Kuopio, Finland; 4 Research Service Unit, Kuopio University Hospital, 70029 Kuopio, Finland; 5 Dasman Diabetes Institute, 15462 Dasman, Kuwai; 6 Center for Life Course Health Research, University of Oulu, 90014 Oulu, Finland; 7 Department of Chronic Disease Prevention, National Institute for Health and Welfare, 00271 Helsinki, Finland; 8 Institute of Public Health and Clinical Nutrition, University of Eastern Finland, 70211 Kuopio, Finland; 9 Department of Ophthalmology, University of Eastern Finland and Kuopio University Hospital, 70029 Kuopio, Finland

**Keywords:** diabetes, intervention, retinopathy, triglycerides

## Abstract

The aim of the study was to find out whether participation in earlier intervention had an effect on the occurrence of retinopathy in study participants. We also examined risk factors (age, sex, weight, fasting and 2 h glucose, fasting insulin, blood pressure, serum lipids) for early retinal changes. The study included 522 individuals (mean 55 years old, range 40–64 years) with impaired glucose tolerance who were randomized into intervention (weight loss, healthy diet, and physical activity, *N* = 265) and control groups (*N* = 257). Intervention lasted for median of four years in 1993–2000, after which annual follow-up visits at study clinics were conducted. In the years 2002–2006 (at least five years after stopping intervention), fundus photography was offered for all study participants in four of five study clinics. Photographs were assessed by two experienced ophthalmologists (A.A. and K.K.), masked for the group assignment. After exclusion of poor quality photographs, the data of 211 individuals (*N* = 113 for intervention and *N* = 98 for control group) were included in the present study. The occurrence of microaneurysms was significantly higher in the control (37/98, 38%) than in the intervention group (27/113, 24%; *p* = 0.029). In the model, including age, sex, diabetes diagnosis before the retinal assessment, body mass index (BMI), and treatment group, the odds ratio for microaneurysms was markedly lower in intervention group (OR 0.52; 0.28–0.97, *p* = 0.039). The only risk factor that predicted the occurrence of microaneurysms was serum triglycerides at baseline (mean ± SD 1.9 ± 0.9 vs. 1.6 ± 0.7, mmol/L, with and without microaneurysms, respectively, *p* = 0.003). Triglycerides associated with decreased microaneurysms in regression analysis for age, sex, fasting glucose, and intervention group (OR 1.92, *p* = 0.018). Lifestyle intervention in overweight and obese individuals with impaired glucose tolerance showed decreased occurrence of retinal microaneurysms. Elevated serum triglycerides were associated to the development of early diabetic microangiopathy.

## 1. Introduction

Diabetes is the major metabolic disorder globally [1]. In 2015, the global prevalence of diabetes was estimated to be 415 million [2]. The number of people with diabetes was predicted to rise up to 642 million in 2040. Prevalence of diabetes is predicted to increase the most in low- and middle-income countries. It has been estimated that five million deaths are caused by diabetes in people aged of 20–79 years.

Approximately 93 million individuals suffering diabetes have diabetic retinopathy (DR) [3]. DR is broadly divided to non-proliferative DR (NPDR) and proliferative diabetic retinopathy (PDR) [4]. The prevalence of any DR is estimated to be 35% and PDR 7% in diabetic patients [3,5]. DR is the fifth most common cause of visual impairment and blindness globally [6]. Globally, DR was the leading cause of blindness among the working-aged population [3,6,7]. Between 1990 and 2010, global visual impairment caused by DR increased from 1.3% to 1.9% and blindness from 2.1% to 2.6% [6].

Characteristic signs of NPDR are microaneurysms, haemorrhages, hard exudates, and intraretinal microvascular abnormalities (IRMA) [4]. In PDR, new detrimental vessels start to grow in different layers of retina that lead to blindness if not treated properly [4]. The major risk factors for DR are duration of diabetes, glycemia, that is, high HbA1c and blood pressure [3]. Risk of DR is higher in type 1 diabetes than in type 2 diabetes [3]. Higher prevalence of diabetic macular edema (DME) is associated with the higher total serum cholesterol and triglycerides [3]. In diabetic patients, cardiovascular disease, previous stroke, and chronic kidney disease increase the risk for vision impairment [8].

In the Finnish Diabetes Prevention Study (DPS) [8], which recruited participants with impaired glucose tolerance (IGT), the lower risk of developing T2D was associated with better insulin sensitivity (IS) and preserved β -cell capacity, probably achieved by changing lifestyles [8,9,10,11]. In this post-hoc analysis, we show decreased retinal microvascular abnormalities in the intervention group of the DPS and suggest that elevated serum triglycerides are associated with early abnormalities of DR. The purpose of the study was to assess whether earlier lifestyle intervention had a beneficial effect on the occurrence of retinopathy.

## 2. Subjects and Methods

### 2.1. Study Design

Five hundred and twenty-two (522) pre-diabetic subjects with IGT participated in the multicenter DPS study in years 1993–1998, and 246 of them were diagnosed with diabetes during the extended follow-up time until 2009 [11]. Originally, 265 subjects were randomly assigned to the intervention group. During the first year, there were altogether seven sessions with a clinical nutritionist. Intervention counselling included healthy dietary choices and increased physical activity. The main goals were reduction in body weight of 5% or more, total fat intake less than 30% of energy consumed, saturated fat intake less than 10% of energy consumed, fiber intake >15 g/1000 kcal, and moderate exercise for 30 min/day or more. Detailed nutritional advices included increased use of whole meal products, vegetables, berries and fruit, low-fat milk and low-fat meat products, soft margarines, and vegetable oils instead of fatty milk products and butter. Dietary advice was based on the three-day food records. Both aerobic and circuit type moderate intensity training were applied. In some centers, group-walking and hiking sessions were also organized. After the first year of intervention, the intervention group visited the study clinics at three-months interval to encourage to permanent lifestyle changes. During the intervention phase, there were altogether a median of 20 sessions per the study participant.

The intervention was most intensive during the first year, followed by a 2–5-year maintenance period with three monthly counselling visits. The other 257 subjects served as controls who received general instruction for lifestyle changes [11]. Detailed information of the study design and interventions has been presented previously [8,11]. All the participants were examined at the yearly basis for their fasting and 2 h glucose at an oral *glucose tolerance test* (OGTT), fasting insulin, blood pressure, and serum lipids. The total time for post-intervention follow-up has been up to 15 years [11].

### 2.2. Examination

**Figure 1:**
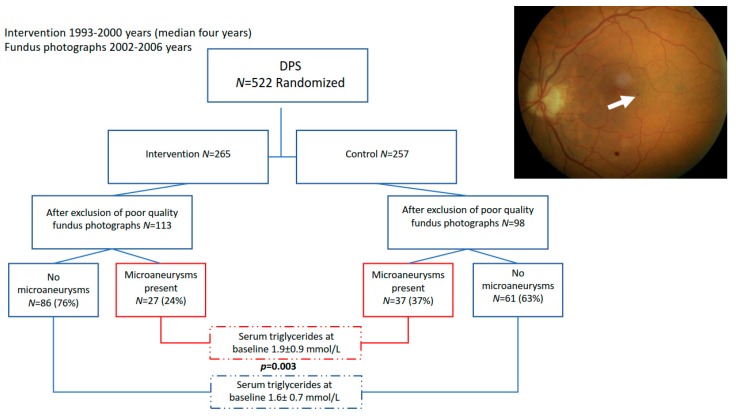
Flowchart of the Diabetes Prevention Study (DPS) for the present ocular examination and baseline serum triglyceride concentrations according to occurrence of microaneurysms (arrow in fundus photograph).

Between the years 2002–2006 (at least five years after intervention phase), the participants in four of the five study centers (excluding the ones who had dropped out) were invited to take part in fundus photography examination (dilated pupils, one field 30-degree). Altogether, 211 subjects went through cross-sectional comprehensive ocular studies where retinal microvascular signs, microaneurysms, hemorrhages, macular edema, soft and hard exudates, intraretinal microvascular abnormality (IRMA), laser scars, and drusen were determined. Altogether, 532 fundus photographs were initially taken from the study population. Photographs that could not be evaluated for every desired parameter were excluded. A total of 145 pictures were excluded from this study as a result of weak quality. The present study group consists of 113 subjects from the intervention group and 98 from the control group (Figure 1). Photographs were assessed for diabetic retinopathy by two experienced ophthalmologists (A.A. and K.K.), and for arteriolosclerotic changes by two ophthalmologists (P.S. and G.v.W.), masked for the group assignment.

### 2.3. Statistical Analysis

Continuous variables were expressed as means with standard deviations and categorical variables as frequencies with percentages. Statistical comparisons were executed by independent samples *t*-test if the variables were continuous; otherwise, Chi-square of Fisher’s exact test was used. Binary logistic regression models were used to study multivariate associations to the risk of microaneurysms. The results from this regression analysis are shown as odds ratios with 95% confidence intervals. All analyses were executed by using IBM SPSS software version 22.0. *p*-value < 0.05 were set to indicate statistical significance results. Statistical analysis was done using Excel 2016 (Microsoft Corp., Redmond, WA, USA) and SPSS Statistics, version 24 for Windows (IBM Inc, Chicago, IL, USA).

## 3. Results

### 3.1. Higher Serum Triglycerides Are Associated with the Occurrence of Microaneurysms

The baseline characteristics of the present study group are shown in Table 1 and Table 2 (data on all 211 study participants as one group are presented in Table 2). Furthermore, Table 1 shows one-year changes in these variables in relation to the presence of microaneurysms (MA) (yes/no). After one-year intervention, individuals in intervention group lost weight (*p* < 0.001) and their fasting glucose (*p* < 0.001), 2 h glucose (*p* = 0.003), and fasting insulin (*p* = 0.001) improved. Moreover, both systolic (*p* = 0.007) and diastolic (*p* = 0.02) blood pressure and serum triglycerides (*p* = 0.001) decreased [11]. The intervention group had less frequently MAs (24%, *p* = 0.029) as compared with the control group (38%) (flow chart, see Figure 1). There was no significant difference in the incidence of other retinal changes identified from ocular photographs between the control and intervention groups (data not shown). Diabetes was developed for 53.1% (34/64) and 52.4% (77/147) in MA and non-MA groups, respectively (*p* = 0.921), during the five-year follow-up. In line with previous DPS studies [8,9,10,11], the control group showed higher risk to develop diabetes 62.2% (61/98) compared with the intervention group (44.2%, 50/113) (OR = 0.48; 0.28–0.84, *p* = 0.009). Next, we analyzed a possible selection bias, but individuals participating in the ophthalmic analyses show similar baseline characteristics compared to non-participants, except for fasting plasma glucose, which was lower in the participants of the present study, suggesting better compliance (Table 2).

**Figure 2:**
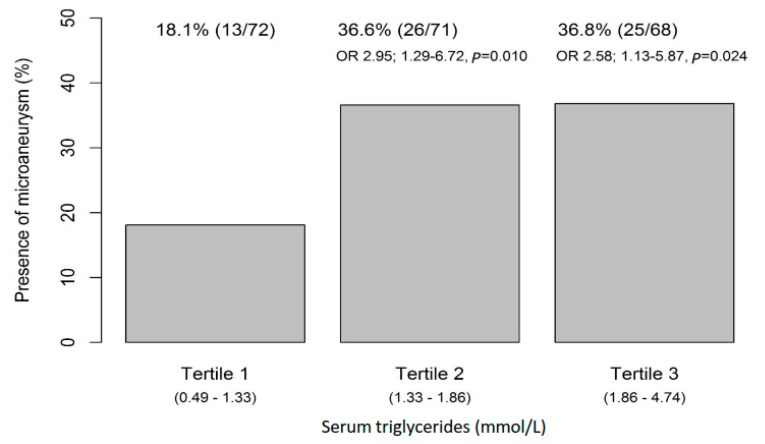
Occurrence of microaneurysms in relation to serum triglyceride tertiles at baseline. OR, odds ratio.

**Figure 3:**
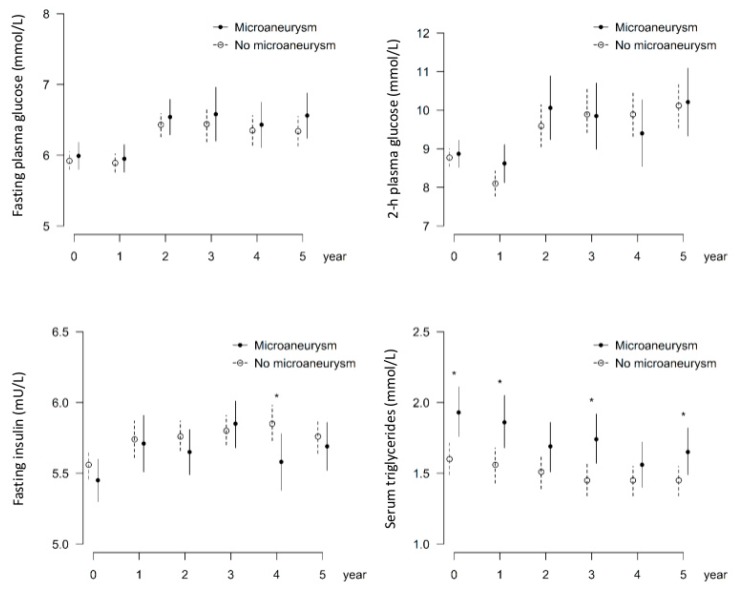
Occurrence of microaneurysms in relation to fasting glucose and insulin, 2 h glucose, and triglycerides. * reveals *p*-values < 0.05. Data from the first five years of follow-up on metabolic variables.

**Figure 4:**
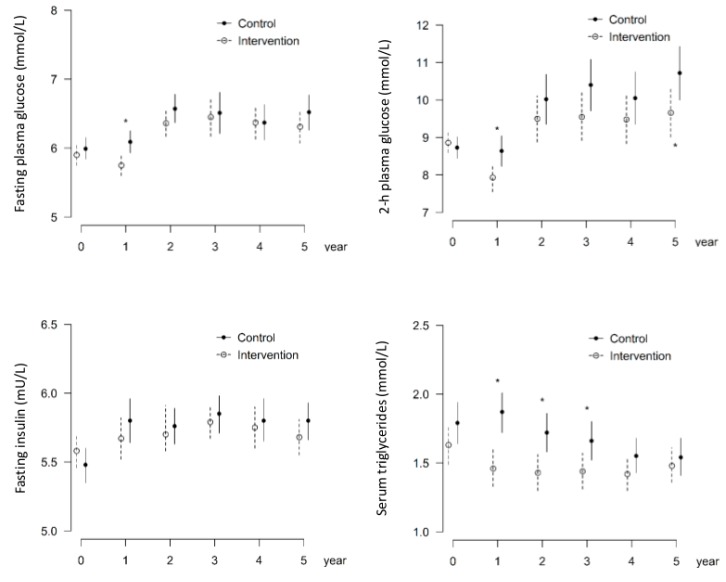
Levels of fasting glucose and insulin, 2 h glucose, and triglycerides in control and intervention study groups. * reveals *p*-values < 0.05. Data from the first five years of follow-up on metabolic variables.

Higher serum triglycerides at baseline were associated with the occurrence of MAs; mean triglyceride values were significantly higher in patients who had MAs at follow-up examination than those who did not (1.9 (0.9) vs. 1.6 mmol/L (0.7), *p* = 0.003) (Figure 1, Table 1). In a tertile analysis, statistically significant associations between MAs and serum triglycerides were observed between the lowest tertile and middle or highest tertiles (36.6%, OR2.95, *p* = 0.010; 36.8%, OR 2.58, *p* = 0.024, respectively) (Figure 2), suggesting an increased risk of MAs with even slightly elevated serum triglycerides. Note that baseline serum triglycerides in the intervention and the control group were at the same level [8]. Next, we analyzed the impact of fasting glucose and insulin, 2 h glucose, and serum triglycerides on the occurrence of MAs by group (Figure 3 and Figure 4) up to five years of follow-up (Figure 4). Triglycerides were the only biomarker that clearly associated with increased MAs during the follow-up, and the life style intervention effect on triglycerides lasted for years (Figure 4).

### 3.2. Intervention Group Shows Decreased Occurrence of Microaneurysms

Finally, the binary logistic regression model for age, sex, incident diabetes, body mass index (BMI), and group confirmed decreased association of the occurrence of MAs in the intervention group (OR 0.52, *p* = 0.039) (Table 3). Furthermore, the impact of average serum triglyceride levels was statistically significant (OR 1.92, *p* = 0.018) (Table 4). Lifestyle intervention effect in this analysis showed a non-significant association, although tendency was protective (OR 0.62, *p* = 0.129). Note that multiple testing for time with relevant post-hoc test was not analyzed.

## 4. Discussion

Our results indicate that an intensive lifestyle intervention for four years in overweight and obese individuals with IGT was associated with decreased appearance of retinal MAs. The only risk factor that predicted the occurrence of MAs was serum triglycerides, whereas fasting or 2 h glucose or blood pressure values did not associate with the appearance of MAs. As far we are aware, this is the first study that shows a connection between lifestyle intervention effects, decreased risk of retinal MAs, and serum triglyceride levels. Former studies reporting lifestyle intervention effects on microvascular complications in individuals with impaired glucose tolerance are scarce. The Diabetes Prevention Program outcome study reveals that lifestyle intervention did not decrease the risk to microvascular (nephropathy, neuropathy, and retinopathy) outcomes [12]. In the China Da Qing Diabetes Prevention Outcome study lasting for six years, carried out in individuals with IGT, combined lifestyle groups showed a 47% reduction in the incidence of severe, vision-threatening retinopathy over a 20-year follow-up [13]. The authors estimated that the reduced incidence of diabetes in the former intervention groups was reason for the less retinopathy incidence. Similar benefits were not seen for nephropathy or neuropathy [13]. The three major risk factors for DR are long-term hyperglycemia, hyperlipidemia, and hypertension [3]. DR is said to be present when MAs appear in the retina, as they are the first clinically seen changes. In our present follow-up of the DPS participants, even slightly elevated serum triglyceride level associated with a higher risk for the development of MAs. This coincides with the conclusion of Chung et al. (2017), that hypertriglyceridemia could be used as a surrogate marker for macular tissue alterations [14]. Our finding also correlates with ACCORD [15] and FIELD [16] studies, where fenofibrate therapy for elevated serum triglycerides reduced the progression of retinopathy and albuminuria.

In addition to MAs, focal arterial narrowing, increased arterial wall reflex, arterial elongation or straightening, arterio-venous ratio or nicking, venous dilatation, tortuosity, or irregularity reveal microvascular arteriolosclerotic introduce complications [17]. The Diabetes Prevention Program outcome study showed that those who did not progress to diabetes had a lower prevalence of total microvascular complications [12]. We did not observe any association between non-incident and incident diabetes and (microvascular) arteriolosclerotic complications. The key question is why in the present study indices of glucose metabolism did not show significant association with the appearance of MAs. We speculate that in the pre-diabetic state, lasting perhaps for many years, glucose values were quite uniform in the whole study population, that is, all had impaired glucose tolerance that contributed to MAs in the whole group, but the impact of lifestyle intervention on glucose values was not that strong that improved glucose tolerance per se would have resulted in the significantly lower frequency of MAs in the intervention group during few years of follow-up. In line with the present study, even slightly elevated serum triglycerides have shown an independent effect on early retinopathy in type 1 diabetes [18]. Furthermore, some other factors modified by lifestyle changes and linked to serum triglycerides could explain the current finding why lifestyle intervention had quite an impressive effect on the occurrence of DR.

As diabetic patients have reduced pressure autoregulation of retinal arterioles and capillaries, the systemic arterial blood pressure is transmitted to the retinal microcirculation [7]. This results in hyperperfusion and increased stress and damage on the retinal capillaries that may evoke the formation of MAs, their leaking, and the development of detrimental diabetic macular oedema [3]. The reduction in blood pressure autoregulation increases with increasing severity of retinopathy. However, we did not observe either in the intervention or control group that blood pressure would be a risk factor to the development of MAs. Probably, this is because of the frequent use of antihypertensive medication [8] and similar blood pressure levels in both intervention and control groups.

Of note, we do not have baseline data on retinal changes. However, there was no selection bias in terms of metabolic variables when comparing the key metabolic and physiological measurements of the current study participants to the rest of the DPS study population, except a lower fasting glucose in the current study (Table 2). Furthermore, intervention and control groups were pretty similar in major baseline measures of importance, and the intervention effect was also similar in the sub-sample to that found in the original study [8]. Therefore, we are confident that the present findings are scientifically important and novel.

Evidence of retinopathy at diagnosis, including the presence of MAs only, significantly increases the risk of retinopathy progression [19,20]. However, improved glucose and blood pressure control reduce the risk of retinopathy, with a linear relationship between the log hazard ratio for retinopathy and both updated mean HbA1c and updated mean blood pressure [21,22]. We should not forget the significance of lifestyle interventions, which have a long-term preventing effect in the progression of pre-diabetes to overt DM or worsening cardiometabolic risk in manifest diabetes [9,11,23,24,25,26,27]. It is well known that weight reduction, healthy dietary choices, and physical activity reduces blood pressure, serum lipids including triglycerides, and improves glycemic control [6]. Prior to drug therapy, life style intervention should be the basis of diabetes prevention and early treatment [8,28,29].

Lifestyle changes emphasizing exercise and healthy diet result in a significant reduction of retinal MAs in pre-diabetes and serum triglycerides may be involved in the development of early retinopathy. Therefore, we suggest intensive lifestyle modification to prevent microvascular complications in patients with pre-diabetes. Triglycerides lowering medication should be considered once there are limitations to carry out non-medical life style intervention [8,11,30,31,32,33].

## Figures and Tables

**Table 1:** Baseline characteristics and one-year changes in these characteristics according to the microaneurysms occurrence.

Variable	No Microaneurysms, *N* = 147	Microaneurysms Present, *N* = 64	*p*-Value
Age, years	54.3 ± 7.3	52.6 ± 6.2	0.083 baseline
Body weight, kg	87.4 ± 14.9 −1.1 ± 6.1	85.1 ± 13.8 −1.0 ± 6.1	0.327 baseline 0.916 change
Fasting plasma glucose, mmol/L	5.9 ± 0.8 0.0 ± 0.7	6.0 ± 0.7 0.0 ± 0.6	0.585 baseline 0.960 change
2 h plasma glucose, mmol/L	8.8 ± 1.4 −0.7 ± 1.9	8.9 ± 1.5 −0.3 ± 2.0	0.676 baseline 0.152 change
Fasting insulin, mU/L	14.1 ± 6.6 −2.0 ± 6.1	14.9 ± 8.9 −1.0 ± 6.2	0.526 baseline 0.282 change
Systolic blood pressure, mmHg	138.6 ± 18.7 −4.9 ± 14.0	136.9 ± 19.0 −2.4 ± 15.2	0.530 baseline 0.238 change
Diastolic blood pressure, mmHg	85.4 ± 9.3 −3.2 ± 8.7	86.5 ± 10.3 −3.2 ± 8.7	0.463 baseline 0.926 change
Serum total cholesterol, mmol/L	5.6 ± 1.0 −0.1 ± 0.6	5.6 ± 0.9 −0.1 ± 0.6	0.532 baseline 0.798 change
High-density lipoprotein cholesterol, mmol/L	1.2 ± 0.3 0.0 ± 0.2	1.2 ± 0.3 0.0 ± 0.2	0.162 baseline 0.691 change
Serum triglycerides, mmol/L	1.6 ± 0.7 0.0 ± 0.6	1.9 ± 0.9 −0.1 ± 0.7	0.003 baseline 0.795 change

**Table 2:** Baseline characteristics of individuals participating in the present study on ocular complications as compared with the non-participants from the original Diabetes Prevention Study (DPS) study population.

Variable	Participants, *N* = 211	No Participants, *N* = 311	*p*-Value
Age, years	53.8 ± 7.0	56.1 ± 7.1	<0.001
Body weight, kg	86.7 ± 14.6	85.8 ± 13.9	0.470
Fasting plasma glucose, mmol/L	5.9 ± 0.8	6.3 ± 0.7	<0.001
2 h plasma glucose, mmol/L	8.8 ± 1.4	9.0 ± 1.5	0.254
Fasting insulin, mU/L	14.4 ± 7.4	15.1 ± 7.5	0.332
Systolic blood pressure, mmHg	138.1 ± 18.8	138.0 ± 16.9	0.968
Diastolic blood pressure, mmHg	85.7 ± 9.6	85.7 ± 9.8	0.985
Serum total cholesterol, mmol/L	5.6 ± 1.0	5.6 ± 0.9	0.642
High-density lipoprotein cholesterol, mmol/L	1.2 ± 0.3	1.2 ± 0.3	0.553
Serum triglycerides, mmol/L	1.7 ± 0.7	1.7 ± 0.8	0.716

**Table 3:** Logistic regression analysis for group comparison. OR, odds ratio; CI, confidence interval; BMI, body mass index.

Variable	OR (95% CI)	*p*-Value
Age, years	0.97 (0.93–1.01)	0.171
Sex, woman	1.60 (0.77–3.33)	0.208
Diabetes, yes	0.80 (0.42–1.54)	0.508
BMI	1.02 (0.96–1.09)	0.523
Group, intervention	0.52 (0.28–0.97)	0.039

**Table 4:** Logistic regression analysis regarding selected variables for the occurrence of microaneurysms.

Variable	OR (95% CI)	*p*-Value
Age, years	0.97 (0.93–1.02)	0.261
Sex, woman	1.86 (0.90–4.05)	0.105
Fasting glucose 0 h	1.04 (0.71–1.49)	0.845
Serum triglycerides, mmol/L	1.92 (1.12–3.35)	0.018
Group, intervention	0.62 (0.33–1.15)	0.129
